# Efficacy of Different Modalities and Frequencies of Physical Exercise on Glucose Control in People with Prediabetes (GLYCEX Randomised Trial)

**DOI:** 10.3390/metabo12121286

**Published:** 2022-12-17

**Authors:** Aina M Galmes-Panades, Miquel Bennasar-Veny, Paula Oliver, Natalia Garcia-Coll, Alice Chaplin, Sergio Fresneda, Laura Gallardo-Alfaro, Carmen García-Ruano, Jadwiga Konieczna, Alfonso Leiva, Lluis Masmiquel, Catalina Pico, Ignacio Ricci-Cabello, Dora Romaguera, Rosmeri Rivera, Pilar Sanchis, Josep Vidal-Conti, Aina M Yañez

**Affiliations:** 1 Global Health and Lifestyle (EVES Group), Health Research Institute of the Balearic Islands (IdISBa), 07120 Palma, Spain; 2 CIBER of Physiopathology of Obesity and Nutrition (CIBEROBN), Instituto de Salud Carlos III, 28029 Madrid, Spain; 3 Research group on Global Health and Human Development, University of the Balearic Islands (UIB), 07120 Palma, Spain; 4 Physical Activity and Sport Sciences Research Group (GICAFE), Institute for Educational Research and Innovation (IRIE), University of the Balearic Islands, 07120 Palma, Spain; 5 Department of Nursing and Physiotherapy, University of the Balearic Islands (UIB), 07120 Palma, Spain; 6 Research Institute of Health Sciences (IUNICS), University of Balearic Islands, 07120 Palma, Spain; 7 CIBER de Epidemiología y Salud Pública (CIBERESP), Institute of Health Carlos III, 28029 Madrid, Spain; 8 Nutrigenomics, Biomarkers and Risk Evaluation (NuBE) Group, University of the Balearic Islands (UIB), 07120 Palma, Spain; 9 Health Research Institute of the Balearic Islands (IdISBa), 07120 Palma, Spain; 10 Research Group on Nutritional Epidemiology & Cardiovascular Physiopathology (NUTRECOR), Health Research Institute of the Balearic Islands (IdISBa), University Hospital Son Espases (HUSE), 07120 Palma, Spain; 11 Research Group in Primary Care and Promotion—Balearic Islands Community (GRAPP-caIB), Health Research Institute of the Balearic Islands (IdISBa), 07003 Palma, Spain; 12 Primary Care Research Unit of Mallorca (IB-Salut), Balearic Health Service, 07002 Palma de Mallorca, Spain; 13 Research Network on Chronicity, Primary Care, and Health Promotion (RICAPPS), Institute of Health Carlos III, 28029 Madrid, Spain; 14 Vascular and Metabolic Diseases Research Group, Health Research Institute of the Balearic Islands [IdISBa], 07120 Palma, Spain; 15 Endocrinology Department, Son Llàtzer University Hospital, 07005 Palma, Spain; 16 Chemistry Department, University of Balearic Islands (UIB), 07120 Palma, Spain; 17 Institute for Educational Research and Innovation (IRIE), University of the Balearic Islands (UIB), 07122 Palma, Spain

**Keywords:** exercise, prediabetes, aerobic exercise, resistance training, type 2 diabetes

## Abstract

To assess the efficacy of different modalities and frequencies of physical exercise on glycaemic control in adults with prediabetes. A two-phase, parallel, randomised, controlled clinical trial will be carried out, in 210 participants. In phase 1, 120 participants will be randomized into four arms: (1) aerobic exercise, (2) aerobic exercise combined with resistance, (3) high-intensity intervallic exercise and (4) control group. In phase 2, 90 new participants will be randomized into three arms, using the exercise modality that showed the best glycaemic control in phase 1 in the following manner: (1) frequency of 5 days/week, (2) frequency of 3 days/week and (3) frequency of 2 days/week. The control group (n = 30) will be included in phase 1 to evaluate the effect of any type of intervention versus no intervention. Data collection will be performed at baseline and after 15 weeks of follow up. Sociodemographic data, medication, comorbidity, blood biochemical parameters, blood pressure, anthropometric measurements, body composition, physical activity, sedentary lifestyle, diet, smoking, alcohol consumption, quality of life and sleep questionnaires will be collected. Physical activity, sedentary behaviour and sleep will be further determined with an accelerometer, and continuous glycaemia will be determined with a glycaemic monitor, both during seven days, at two time points. The main dependent variable will be the reduction in the mean amplitude of glycaemic excursions. The impact of these interventions on health will also be evaluated through gene expression analysis in peripheral blood cells. The results of this study will contribute to a better understanding of the mechanisms behind the glucose response to physical exercise in a population with prediabetes as well as improve physical exercise prescriptions for diabetes prevention. Increasing glycaemic control in people with prediabetes through physical exercise offers an opportunity to prevent diabetes and reduce associated comorbidities and health costs.

## 1. Introduction

Type 2 diabetes mellitus (T2D) is a highly prevalent chronic disease causing significant complications that reduce the quality of life and life expectancy [1]. Type 2 diabetes also represents a considerable economic burden for health systems and society and has become one of the major worldwide public health problems. In addition, T2D and its risk factors (obesity, diet and physical inactivity) affect the most socially vulnerable population to a greater extent, contributing to social inequalities in health [2].

The development of T2D is gradual and preceded by a prediabetic state [3]. The American Diabetes Association (ADA) defines prediabetes as fasting plasma glucose (FPG) levels between 100 and 125 mg/dL, and/or a glucose tolerance test (GTT) after 2 h between 140 and 199 mg/dL, and/or glycated haemoglobin (HbA1c) levels between 5.7 and 6.4% [3]. The International Diabetes Federation (IDF) estimated that 7.5% of the population worldwide aged 20–79 years has prediabetes [2]. However, the prevalence in the Spanish population is close to 14% [4]. Furthermore, an ADA expert panel estimated that 70% of the population with prediabetes will develop T2D in the future [5], with an annual incidence of 5–10% [6]. Nevertheless, prediabetes can revert to normoglycaemia, especially with the implementation of lifestyle interventions [7,8]. For the population with prediabetes, a lifestyle modification focused on a healthy diet [9] and physical activity (PA) promotion [10] is the cornerstone of prevention [7].

Most studies evaluating different exercise modalities and frequencies use only point measures such as HbA1c, FPG or other indicators of glucose metabolism [11,12,13]. The mean amplitude of glycaemic excursions (MAGE) and percentage of time in range (TIR) are careful measures of glycaemic control obtained by continuous glucose monitoring (CGM) [14]. Fluctuations in blood glucose levels during the day likely carry greater health risks than FPG levels or HbA1c [15]. Several studies suggest that physical exercise reduces the postprandial glucose response [16,17] and reduces the MAGE [18]. However, few studies have examined the effect of exercise on glycaemic variability using CGM (24 h/d) [19,20].

In addition to more classical approaches, transcriptomic analysis in blood cells is increasingly used for clinical purposes to identify early risk biomarkers of different pathologies, including T2D [21]. Peripheral blood cells (PBC), which include the peripheral blood mononuclear cell (PBMC) fraction, can express almost the entire genome and mimic gene expression profiles of internal organs and tissues [22]. These cells can reflect metabolic alterations related to increased adiposity and/or obesity early on, as well as metabolic recovery after a weight-loss intervention [23]. Moreover, PBMC transcriptomic profile is affected by exercise [24]. For all these reasons, these blood cells are an interesting non-invasive biological source of biomarkers of the impact of exercise on homeostatic glucose control.

Current evidence shows that preventive interventions focused on lifestyle modification (PA and diet) are efficient in clinical practice with the prediabetic population [25]. According to the World Health Organization (WHO) recommendations, between 150–300 min of physical exercise per week should be accumulated [26], but it is unknown what time distribution is most beneficial for preventing T2D. Furthermore, there are few studies comparing the effect of different modalities of physical exercise (such as aerobic or resistance) on glycaemic control and none have adequate sample sizes to allow generalisability of their results [27]. Some studies on the effect of different modalities and frequencies on visceral adipose tissue (VAT) reduction suggest that aerobic training (AT) should be practiced 3 days/week, with 30–60 min per session. In the case of high-intensity interval training (HIIT), even sessions of less than 30 min would achieve sufficient energy expenditure to produce effects [28].

Physical activity can be classified into four dimensions: modality, frequency, duration and intensity. Modality is the type of PA performed (aerobic, anaerobic, resistance or balance); frequency refers to the number of sessions/day or week; duration refers to the amount of time (minutes or hours) spent performing PA; and intensity is an indicator of metabolic demand of the activity. The higher the intensity, the greater the effort required to perform the activity. Intensity can be quantified objectively with physiological measurements (i.e., heart rate or oxygen consumption), measured subjectively (i.e., talk test or Borg scale) or quantified by body movements (i.e., body accelerations in three axes) [29].

Aerobic training is characterised by training at an intensity that allows the aerobic route to be used for obtaining energy, in which the body’s large muscles move rhythmically for a sustained period of time. Aerobic training has been associated with glucose control and improvement in insulin sensitivity [30]. This type of exercise can also be called aerobic activity or endurance activity and improves cardiorespiratory fitness. Some examples of AT are running, brisk walking, swimming or cycling [26].

Interval training (IT) is a modality of physical training that consists of short periods of exercise of varying intensity with short periods of rest in between. This modality of exercise strategy is associated with glycogen depletion, which may induce improvements in insulin sensitivity [31]. High-intensity interval training is a type of interval training that is performed at high intensity. It is a very vigorous physical activity, with a heart rate ≥85% or a VO_2_Max ≥60% [32], performed in short bursts interspersed with breaks [33]. There is a consensus regarding the decrease in training time in the HIIT exercise modality [19,28,34]; because it is a vigorous activity, 75 min/week is recommended, instead of 150 min/week in the case of moderate activities [26].

Resistance training (RT), also referred to as muscle strengthening, is defined as the exercise performed against some type of resistance to increase muscle strength, muscle endurance or muscle power. Resistance training exercises can be performed by weightlifting using machine weights, free weights or elastic bands that resist movement. Some examples of RT are bench press, seated row, shoulder press, leg press, or weight strength [35]. Resistance training regimens should include multi-joint exercises that affect more than one muscle group (i.e., lower back extension, chest press, shoulder press, pull-down, dips, leg press or squats) and involve major muscle groups, such as quadriceps extensions, leg curls, biceps curls and triceps extensions [36,37]. Research findings show that this intervention strategy increases insulin sensitivity and glucose tolerance primarily through increased skeletal muscle mass [37].

Although numerous studies have shown the beneficial effects of physical exercise on glycaemic control in people with prediabetes [19,38,39], there are few studies comparing different modalities of physical exercise (i.e., AT, RT, IT). Furthermore, none of the studies conducted included an adequate sample size to generalise the results [11,27]. One study that compared moderate AT and HIIT reported greater benefits of AT on body mass index (BMI) and body fat, whereas similar results were obtained from both modalities for FPG and HbA1c [11]. Another study, also conducted on people with prediabetes, concludes that both RT and AT lead to a decrease in insulin resistance. However, maximal RT reported a greater increase in glucose uptake capacity (in muscles), whereas AT showed a greater ability to increase insulin sensitivity, which supports the recommendation to combine maximal RT with AT to prevent the progression of prediabetes to T2D [27].

In summary, although there is a wide amount of evidence showing the beneficial effects of PA on health, and particularly, on the prevention of T2D, there is no clear consensus on the specific type of exercise to be prescribed to achieve the maximum benefits. This project aims to explore different types of exercise and different frequencies, to understand their impact on glucose homeostatic control, by using new technological approaches.

Most studies agree that interventions of at least 12 weeks should be carried out to assess changes in blood glucose, with a training frequency of 3 days/week [12,13,38,40]. Likewise, HbA1c reflects the mean glucose concentration over the previous 8–12 weeks [41]. This experimental study has been designed to evaluate the effect of different modalities and frequencies of physical exercise in participants with prediabetes at 15 weeks of follow-up, considering in all the experimental groups the amount of time recommended by WHO.

Furthermore, this project will use CGM, which will provide us with very accurate data on glycaemic variability throughout the day and relate them to daily activity data (PA and sedentary behaviour (SB), sleep, diet intake, among others), allowing us to determine new factors involved in glycaemic control that may suggest new forms of intervention in prediabetes.

## 2. Objectives

### 2.1. Main Aim

The main aim of the present study is to compare the efficacy of three modalities (Phase 1) plus control group and three different frequencies (Phase 2) of physical exercise on MAGE in adults with prediabetes.

### 2.2. Secondary Aim

The secondary aims are to evaluate the effect of different physical exercise modalities and frequencies on the following variables: FPG, glycaemic variability measured continuously (24 h), normalization of glycaemic values (reversal), BMI, body composition (percentage and grams of total fat mass and muscle mass), visceral adipose tissue (VAT) waist circumference, lipid profile, inflammation markers, blood pressure, sleep duration and quality and transcriptomic markers of metabolic health assessed in PBC.

## 3. Experimental Design

### 3.1. Design

The GLYCEX study (GLYcaemic Control with EXercise) is a randomised, parallel, phase II clinical trial with three active arms in each of the two phases plus a control group in the first phase. The intervention groups will receive a supervised exercise intervention for 15 weeks in both phases. The control group will receive general recommendations of PA, based on WHO recommendations, and after 15 weeks, will be invited to participate in the second phase of the study. Baseline evaluations will be repeated after 15 weeks.

Study interventions were reported using the Template for Intervention Description and Replication (TIDieR) as a reference [42] (Supplementary Material Table S1). The present protocol was elaborated following the Standard Protocol Items: Recommendations for Intervention Trials (SPIRIT) 2013 Statement [43]. Reporting of the study will follow the CONSORT statement recommendations on RCTs [44]. Table 1 summarizes all items included in the trial registry, as suggested by the World Health Organization (WHO, 2018).

### 3.2. Participants

Participants’ identification and recruitment will be carried out at different primary care centres from Mallorca, Spain, that formally agree to participate in this project, and through posters distributed at different points of interest, such as hospitals, universities, primary care centres and patient associations. Likewise, it will also be disseminated through social networks, such as Twitter. Interested patients will receive the information sheet, sign the written informed consent and eligibility criteria will be verified.

Inclusion criteria: Adults aged 18–65 years, with overweight or obesity (BMI >25 and <35 Kg/m^2^), inactive (<150 min PA/week) and with prediabetes (fasting blood glucose 100–126 mg/dL), and who have signed the informed consent will be included.

Exclusion criteria: People with uncontrolled hypertension, diagnosis of T2D or oral anti-diabetic prescription, active cancer, terminal illness or cognitive impairment, pregnancy, cardiovascular disease, inability to perform moderate-vigorous physical exercise for the next 3 months, major surgery or hospital admission in the last 3 months, haematological disease that interferes with HbA1c determination, presence of any condition (medical, psychological, social or geographical) current or anticipated that limits participation in the study, or participation in another clinical trial will be excluded.

### 3.3. Sample Size and Randomization

The sample size has been calculated to detect significant differences in MAGE of at least 1.5 mg/dL and considering a standard deviation of 1.4 mg/dL [45] in any of the three modalities and a dropout rate of 20%, so 30 subjects will be needed in each group, with a sample of 180 participants, 90 in each phase. Furthermore, a control group (30 patients) will be added to evaluate the effect of any type of monitored exercise in these patients versus usual clinical practice. Randomization will be carried out by permuted blocks of eight in a 1:1:1:1 ratio and stratified by prognostic factors: gender, age and obesity using the open-source Oxford Minimization and Randomization (OxMaR) program [46].

## 4. Procedure

### 4.1. Description of Interventions

**Figure 1:**
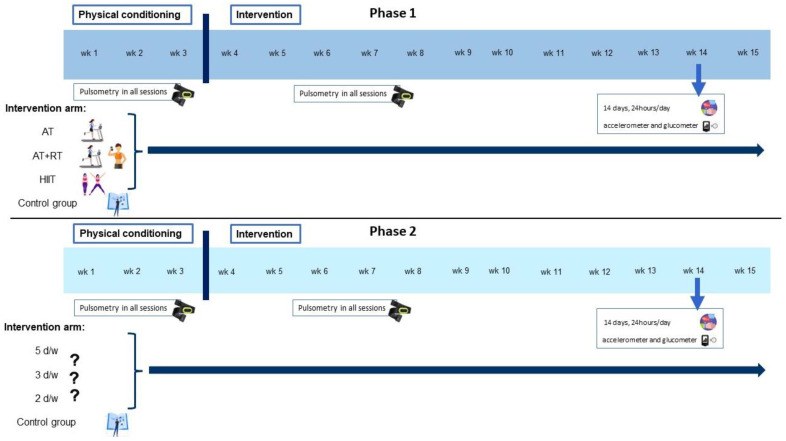
Intervention description. Abbreviations: AT, aerobic training; RT, resistance training; HIIT, high-intensity interval training; d/w, days/week; wk, week.

The intervention will be designed and implemented by a sports science professional. All groups in both phases will perform a minimum of 150 min of moderate physical exercise per week or a minimum of 75 min of vigorous exercise per week, always performed under the supervision of a sports science professional. During the intervention participants will progress to 300 min of moderate PA or 150 min of vigorous PA. Prior to the start of the full intervention, participants will undergo a 3-week pre-intervention physical conditioning. Each intervention will last 12 weeks (see Figure 1). Before starting the intervention program, participants will be referred to a physician for medical testing and clearance. During all physical exercise sessions, participants will wear a heart rate (HR) device that will relay their HR to a laptop, from which the sports scientist will be able to control the intensity of the session. The heart rate monitor model to be used is the Polar OH1, which has been validated in previous studies [47]. Polar HR monitors have demonstrated a high level of agreement with the electrocardiogram and can therefore be used as a valid measure of HR in both laboratory and field studies to measure HR during moderate-to-vigorous physical activity (MVPA) [47]. Heart rate will only be measured during the training sessions, with the aim to ensure that the intensity at which the participant performs the exercises is appropriate according to the protocol (it will vary for each intervention arm). Adherence to the intervention will be measured by attendance at the training sessions that make up the intervention.

#### 4.1.1. Phase 1

Three exercise modalities will be compared: (1) AT, (2) AT combined with RT and (3) HIIT. During all intervention sessions, participants will wear an HR monitor connected to a computer. In this way, study staff will be able to control the intensity of the session and individualise exercise prescriptions in real time. Heart rate monitoring will be a key element in ensuring that each exercise is performed at the target intensity, by calculating the intensity at which each volunteer performs the exercise based on a percentage of their HR. Despite there being numerous formulas for estimating the maximum heart ratio (HRmax), studies based on meta-analysis reported that [HRmax = 208 − 0.7 × age] is a more reliable option to predict HRmax [48,49]. Once the HRmax has been estimated, the Karvonen formula will be used to calculate the HR corresponding to a given percentage of intensity. For example, if we want a subject to exercise at 60% of their HRmax, it will be calculated using the Karvonen formula, which takes into account HRmax and basal heart rate (HRB), as follows: HRB + % intensity desired [HRmax—HRB] [50]. This formula has been recommended for both people with cardiac diseases and athletes due to the accurate characteristics of the formula [50].

1) Aerobic Training intervention (AT): Perform 50 min/day, 3 days/week, totalling 150 min/week [34,51] at moderate intensity, as recommended by WHO [26], in a range of 65–75% HRMax. Because any form of aerobic exercise involving large muscle groups and causing sustained increases in HR is likely to be beneficial [52], the type of aerobic exercise will be agreed on with each group of patients, varying from four to eight people. There will be a choice of exercises and participants will be able to choose a combination of up to two different exercises. The participants’ choice of the type of activity to be performed is expected to encourage greater adherence. The range of exercises will be brisk walking or running, swimming and/or aerobic dancing.
2) Aerobic Training plus Resistance Training intervention (AT+RT): Perform 50 min/day, 3 days/week, starting with 50% of 1-repetition maximum (1-RM) and follow a progression of increasing loads up to 75% of 1-RM for optimal gains in strength and insulin action [51]. In each session, between five and ten exercises will be worked on, performing 10–15 repetitions, and progressing to 8–10 lifting as the weight increases, involving the major muscle groups from the core, lower body and upper body [53,54]. In all sessions there will be a 3 min warm-up at the beginning of the session and a 2 min cool-down at the end of the session [34].
3) High Intensity Interval Training intervention (HIIT): To be considered high intensity, the heart rate needs to be above ≥85% [19,34,55]. Perform 25 min/day, 3 days/week, totalling 75 min/week [34,51] at a vigorous intensity, as recommended by WHO [26]. Starting with four intervals lasting 1 min keeping in a range of 85–90% HRMax, separated by 1 min of low intensity activity (no static) (4 × 1 min intervals), a progression will be followed by increasing the number of circuits, up to ten (10 × 1 min intervals) [19,55,56]. In all sessions there will be a 3 min warm-up at the beginning of the session and a 2 min cool-down at the end of the session [19,34,55,56]. Although the target population is a sedentary population, we expect the HIIT approach, despite being high intensity, to be well received, due to its growing popularity, as demonstrated by a study in which 62% of inactive participants preferred HIIT to other types of exercise [57].
4) Participants in the control group will receive written standard PA recommendations in this phase.

#### 4.1.2. Phase 2

Different exercise frequencies per week will be compared using the modality that best controlled blood glucose in phase 1: (1) five sessions/week; (2) three sessions/week and (3) two sessions/week. As mentioned above, all groups in both phases will perform a minimum of 150 min of moderate physical exercise per week or 75 min of vigorous exercise per week, always performed under the supervision of a sports science professional.

### 4.2. Data Collection and Procedures

Data collection (visit −1 and 1) will be performed at the Health Research Institute of the Balearic Islands (IdISBa). Visit 0 and the intervention sessions will be held at the facilities of the University of the Balearic Islands.

In both phase 1 and phase 2, the same number of data collection visits will be carried out and the same information will be collected at each of the visits (Table 1).

#### 4.2.1. Visit −1 (V−1)

Before inclusion in the study, potential participants will be scheduled for a first visit to verify inclusion and exclusion criteria. During this visit, informed consent and baseline data will be collected including sociodemographic data, medication and concomitant pathologies, tobacco and alcohol consumption, assessment of PA, SB, diet, quality of life, quality of sleep, anthropometric and body composition parameters, blood pressure, biochemical blood analysis and gene expression analysis in blood cells. Participants meeting all eligibility criteria will be randomly assigned, stratified by sex, age and obesity, to one of the three intervention groups or control group. The same procedure will be followed for both phases of the study. All participants will be asked to wear an accelerometer, a continuous blood glucose monitor and a daily activities e-diary (intake, sleep, SB and PA) for 14 days to collect accurate baseline PA and blood glucose data.

#### 4.2.2. Visit 0 (V0):

Participants will be informed of the exercise modality or frequency to which they have been assigned and the days on which the physical exercise sessions will take place will be agreed upon. During the visit, the participant will wear a heart rate monitor to record their resting heart rate.

#### 4.2.3. Visit 1 (V1)

Before the follow-up visit (week 15 of intervention), the accelerometer and the continuous glucose monitoring biosensor will be placed for 14 consecutive days, 24 h/d, and participants will complete the e-diary during these 14 days. At the end of follow-up (15 weeks), the accelerometer and continuous glucose monitoring biosensor will be removed. During this visit medication and concomitant pathologies, assessment of PA, SB, diet, tobacco and alcohol consumption, assessment of the quality of life and quality of sleep, anthropometric and body composition parameters, blood pressure, biochemical blood analysis, gene expression analysis in blood cells and adverse events will be collected for all participants (see Table 1).

### 4.3. Data Collection

#### 4.3.1. Biological Samples and Laboratory Procedures

At visits V−1 and V1, venous blood samples will be collected after an overnight fast of ≥8 h. Blood tests performed will include FPG, HbA1c, total cholesterol, high-density lipoprotein cholesterol (HDL-c), low-density lipoprotein cholesterol (LDL-c), triglycerides (TG), gamma-glutamyl transferase (GGT), aspartate aminotransferase (AST), platelets, leukocytes, high-sensitivity C-reactive protein (hsCRP), interleukin-1 (IL-1), interleukin-6 (IL-6), interleukin-8 (IL-8), advanced glycation end products (AGEs), adiponectin and leptin. Blood samples will be analysed centrally. The TG-glucose index (TG) will also be calculated [58]. Additionally, the expression of relevant genes indicative of metabolic health previously identified [59,60], as well as selected genes with a key role in the pathogenesis of T2D will be assessed by real-time qPCR in blood cells.

#### 4.3.2. Glycaemic Variability

The continuous blood glucose monitoring system (Dexcom G6, Dexcom Inc., San Diego, CA, USA) [61] will be used to assess glycaemic variability for 14 consecutive days in all samples [62]. The sensor will be placed at V−1 and removed after 14 days. It will be placed again at the beginning of week 14 and removed at V1. The Dexcom G6 sensor will be inserted into the subcutaneous adipose tissue in the lower abdomen (below the umbilicus) [19,63]. To optimise the usefulness of continuous glycaemic monitoring, participants will be asked to keep an electronic diary (e-diary), where they will record the food intake, the timing of intakes and the time they go to sleep and wake up during 14 consecutive days. Mean amplitude of glycaemic excursions and time in range (TIR) will be estimated from the 14-day sensor glucose profiles. Mean amplitude of glycaemic excursions will be calculated by taking the mean of the increases or decreases in blood glucose (from nadirs to peaks or vice versa) when the rising and falling segments exceed the 1 standard deviation blood glucose value over a 24 h measurement period [14].

#### 4.3.3. Accelerometer

To quantify PA, SB and sleep, participants will wear a wrist accelerometer on the non-dominant arm for 14 days, 24 h a day (V−1 and V1) [64], and will complete a sleep log. The device (GENEActiv, ActivInsights Ltd., Kimbolton, UK) is a tri-axial accelerometer that allows the following information to be obtained: sedentary time, light PA, moderate PA, vigorous PA and sleep time [65]. The raw data files will be processed with the R package (R Core Team, Vienna, Austria) using the open-source R package GGIR, version 1.2–5 (cran.rproject.org/web/packages/ggir/index.html, access date: 15 December 2022), which has been validated against the self-calibrated functions [66].

#### 4.3.4. Diet

At visits V−1 and V1, diet quality will be assessed using the validated 17-item MedDiet adherence questionnaire [67]. Each item is scored 1 (adherence) or 0 (non-adherence), so the total score can range from 0 to 17, with 0 indicating no adherence and 17 indicating maximum adherence. Moreover, as explained in the glycaemic variability section, to determine the quality of the diet beyond adherence to the Mediterranean diet, and its effects on glycaemic variability, other questions related to diet will be included in the e-diary about the time of each intake and the type and quantity of food intake during 14 consecutive days at baseline and follow-up.

#### 4.3.5. Physical Activity and Sedentary Behaviour

At visits V−1 and V1, the validated REGICOR PA questionnaire for adults, which measures PA levels over the previous 12 months [68], and the validated SB Nurses’ Health Study (NHS) questionnaire for the Spanish population [69] will be administered. The REGICOR PA questionnaire is a sensitive tool to detect moderate and vigorous PA changes in epidemiological studies. It includes a total of 12 questions divided into three categories: questions on the type and intensity of leisure-time physical exercise (walking, brisk walking, walking in the countryside, gardening and climbing stairs), occupational PA and SB, including time spent sleeping. The average daily energy cost of PA is then calculated and expressed in metabolic equivalents (METs) [70]. The REGICOR PA questionnaire also collects data on the average monthly time spent in light (<4 METs), moderate (4–5.5 METs) and vigorous (≥6 METs) PA. Finally, the number of weekly hours of SB is also recorded [68]. The number of hours spent on SB will be recorded using the NHS questionnaire. The types of activities recorded are watching TV, sitting in front of a computer/screen, sitting during transport as a driver or passenger and total sitting time. The frequency of activities is assessed as times in minutes (<30 min; between 30 and 60 min) or hours (between 1 and 9 or more) on an average day, both on weekdays and weekends separately [69].

#### 4.3.6. Anthropometric Measurements

Height, body weight, BMI, waist circumference (WC) and body composition (bioelectrical impedance; Tanita BC-418; Tanita Corp., Tokyo, Japan [71]), will be obtained at visits V−1 and V1 (except for height, which will be measured only at V−1). All anthropometric measurements will be collected according to the recommendations of the International Standards for the Anthropometric assessment (ISAK) [72]. Furthermore, all measurements will be performed by well-trained technicians or researchers to minimize coefficients of variation. Height will be measured to the mm with a book-foot stadiometer with participants standing barefoot and with the head placed in the Frankfort plane; body weight will be measured to the nearest 0. 1 kg with a Tanita body-fat analyser, which will also measure body composition by bioelectrical impedance; BMI will be calculated using the standard formula (weight (kg)/height (m^2^)); and, using a tape measure, WC will be taken at the narrowest point between the lower costal rib and the iliac crest. During height and weight measurements, participants shall adopt a relaxed standing position, with arms crossed over the chest, feet together on the floor and buttock muscles relaxed; both measurements will be taken twice, and the median value will be considered for statistical analyses.

#### 4.3.7. Blood Pressure

At visits V−1 and V1, consecutive blood pressure measurements will be taken in each arm and recorded. The arm with the highest median blood pressure shall be considered for statistical analysis and subsequent measurements during follow-up shall be taken from this same arm (V1). If the median blood pressures of the two arms are identical, subsequent measurements shall be taken from the non-dominant arm. Blood pressure measurements will be taken at the brachial artery after 5 min of rest in a seated position with a validated oscillometer (Omron M3). For statistical analyses, the mean of two measurements taken 2 min apart will be considered.

#### 4.3.8. Quality of Life and Sleep

Quality of life and sleep quality will be assessed at V−1 and V1, using the following validated questionnaires: The European Quality of Life-5 Dimensions (EuroQol-5D) questionnaire is a widely used measure of self-reported health-related quality of life. The questionnaire is divided into two parts. The first comprises five domains: mobility, personal care, usual activities, pain/discomfort and anxiety/depression. For each domain, participants can indicate the level of problems experienced on a three-point categorical response scale (no problem, some problems or severe problems). From the answers given, a single summary score is created, which indicates the self-reported health status. The second part is a Visual Analogue Scale, which captures the overall health perceived by the participant in a score ranging from 0 (worst imaginable health) to 100 (best imaginable health) [73]. The six-item Medical Outcome Study Son Index (Bocado-Sleep) is the shortened version of the 12-item MIS-Sleep scale [74,75]. It is a validated instrument used to assess the quality and quantity of self-reported sleep during the previous month. It uses a list of six items divided into three different domains: sleep disturbances, daytime sleepiness, sleep adequacy, and awakening with shortness of breath or headache. The items are scored on a six-point scale ranging from all the time to never. For interpretation, the direct scores are transformed into a scale from 0 to 100, with no cut-off points; the higher the score, the greater the intensity of the item assessed.

## 5. Results

### 5.1. Main Dependent Variable

The primary outcome variable is the change in MAGE at 15 weeks of follow-up.

Secondary dependent variables are FGP, TIR, HbA1c, TG, blood pressure, body composition, WC, transcriptomic biomarkers in blood and quality of life.

### 5.2. Main Independent Variable

The main independent variable is the assigned group.

### 5.3. Statistical Analysis

Statistical analyses will be performed using R v3.3.1 and the Stata v17.0 program. *p*-values <0.05 will be deemed statistically significant.

- Descriptive analysis, labelling and data cleaning: Assessment of outliers and extreme values, detection and labelling of missing and/or non-applicable values, and description of the distribution of each of the variables. Normality test and scatter plots.

- Descriptive baseline analysis: Sociodemographic characteristics and MAGE and FPG by the different intervention modalities at each phase.

- Main analysis: This will be different for each of the phases and the main analysis will be performed by intention-to-treat. In the first phase, a comparison of MAGE and FPG between the four modalities will be made, and in the second phase the three frequencies will be compared. The ANOVA (GLM) test will be used adjusting by baseline values for both phases. If the assumptions of normality are not met, the Kruskal–Wallis test will be applied. A further adjustment for baseline characteristics, if necessary, will be performed using GLM to determine the effect of membership in each exercise group on decreasing MAGE and FPG. A similar analysis will be performed for each of the secondary dependent variables (HbA1c, TG, blood pressure, body composition, waist circumference and quality of life). Missing values will be replaced using the multiple imputation model (MICE) [76]. Before starting the analysis, a review of possible outliers and range checks for data values will be performed.

### 5.4. Ethical Considerations

The GLYCEX study will follow the Declaration of Helsinki ethical standards, and all procedures will be approved by the Institutional Review Board of the Balearic Islands Health Service Research Ethics Committee (CEI-IB). The study has been registered on clinicaltrials.gov with the ID NCT05612698 on 4 November 2022. In the event of substantial modifications to the protocol, the Ethics Committee will be informed. All participants will sign a written informed consent, and all data collected will be kept anonymous and confidential. Participants may voluntarily withdraw their informed consent at any time during the study. Data collected prior to patient withdrawal will be retained and used by the study investigators as specified in the informed consent. All participants, before starting the intervention, will undergo a medical examination to certify that they are able to perform physical exercise. No harm or adverse effects are expected given the characteristics of the intervention; however, injuries, such as blows or sprains, derived from the exercise practice, which in no case would suppose a serious risk to the health of the participants, could occur. However, adverse effects, if any, will be recorded and communicated to the CEI-IB.

### 5.5. Validity and Reliability

The present study will provide important evidence on the effect of supervised exercise interventions on glycaemic control in a population with prediabetes. The GLYCEX study will include a sample of participants with prediabetes and will use validated measurement to determine the intensity and duration of exercise both in free-living environments and during the intervention sessions, as well as measurements of glucose levels at specific times (fasting blood analysis) and with continuous glucose monitors. All these measurements will allow us to obtain robust results that can be extrapolated to the population. Internal validity will be warranted by a randomization process based on allocation sequence generation, blinded to the PI and staff involved in the intervention. Moreover, the data analyst and the PI will be blinded to patient allocation to reduce biases in the evaluation of the intervention. Lastly, the interventions are based on lasted scientific evidence and official guidelines.

## 6. Discussion

The present study will provide information on the efficacy of a supervised physical exercise intervention to reduce FPG in people at risk of T2D, allowing us to determine which exercise modality, and with what weekly frequency, is the most appropriate for a population of adults with prediabetes. Furthermore, this information will be of great use to the scientific community and the general population to increase knowledge about prediabetes reversion.

The GLYCEX study presents some limitations. First of all, any change in habits implies an effort, and therefore, attendance at the intervention sessions may not be as high as desired (more than 80%), which could make it difficult to analyse the effect of the intervention. However, as the sessions are supervised by a sports professional, with small groups and personalized adaptations, it is expected that the volunteers will have better adherence. Secondly, self-reported questionnaires are often based on participants’ statements, with the biases that this may entail. Nevertheless, this study will use validated questionnaires which will not penalize the quality of the results, as well as other objective measurements, such as bioelectrical impedance, blood tests, continuous glucose monitoring, pulsometers and three-axial accelerometers. Thirdly, evaluating the effect of the intervention under experimental conditions compromises the generalization of results in other conditions. Finally, patient recruitment can be difficult and time-consuming. To avoid this limitation, recruitment through primary care professionals will be combined with posters distributed in strategic locations and social network publicity to recruit volunteers.

A strength of the proposed intervention is the personalized approach, which considers the patient’s preferences, giving a choice between different types of aerobic exercise, in two of the three intervention arms of the first phase.

Finally, the results of our study could contribute to better design strategies for the reversion of prediabetes to normoglycemia. Given the increasing prevalence of prediabetes and T2D and the associated costs, determining interventions that could control glycemia in people with prediabetes could be a useful approach to palliate the actual T2D pandemic.

## Figures and Tables

**Table 1:** Data collection by visits.

	Initial Evaluation	Start	
Visit number	−1	0	1
Time	−Day 7	Day 0	Week 15
Informed consent	X		
Inclusion/exclusion criteria	X		
Randomization	X		
Blood sample	X		X
Glucose monitoring	X		X
Baseline data	X		
Cites management		X	
Adverse events			X
Anthropometric measurements	X		X
Blood pressure	X		X
Physical activity questionnaire	X		X
Sedentary behaviour questionnaire	X		X
Diet, alcohol and smoking questionnaire	X		X
Quality of life questionnaire	X		X
Accelerometer	X		X
Pulsometer		X	

## Data Availability

Not applicable.

## Supplementary Materials

The following supporting information can be downloaded at: https://www.mdpi.com/article/10.3390/metabo12121286/s1, Supplementary Table S1:_TIDieR author tool.
