# Supplementary material for: Efficacy of Different Modalities and Frequencies of Physical Exercise on Glucose Control in People with Prediabetes (GLYCEX Randomised Trial)
Source: Metabolites. 2022 Dec 17;12(12):1286. doi: 10.3390/metabo12121286 (PMC9785307; doi:10.3390/metabo12121286)
Supplement: Supplementary file 1 [file metabolites-12-01286-s001.zip › metabolites-2088977-Supplementary.pdf]

TIDieR

GLYCEX study (GLYcaemic Control with EXercise)

Why:

Although numerous studies have shown the beneficial effects of physical exercise on glycaemic control in people with prediabetes, there are few studies com-paring different modalities of physical exercise (i.e. AT, RT, IT). Furthermore, none of the studies conducted has included an adequate sample size to generalise the results. One study comparing moderate AT and HIIT reported greater benefits of AT on body mass index (BMI) and body fat, but similar results on FPG and HbA1c. Another study, also conducted on people with prediabetes, concludes that both RT and AT lead to a decrease in insulin resistance; however, maximal RT reported a greater in-crease in glucose uptake capacity (in muscles), whereas AT showed a greater ability to increase insulin sensitivity, which supports the recommendation to combine maximal RT with AT to prevent the progression of prediabetes to T2D. In summary, although there is a wide amount of evidence showing the beneficial effects of PA on health, and particularly, on the prevention of T2D, there is no clear consensus on the specific type of exercise to be prescribed to achieve the maximum benefits.

What (material):

In addition to the patient information sheet and a copy of the informed consent form, various sports equipment will be used during the intervention sessions, such as: weightlifting machines, mats, dumbbells, swimming pool facilities, aerobic dance rooms, pulsometers, accelerometers, bioimpedance, among others.

What (procedures):

The intervention is composed of 2 phases. In the first phase participants will be randomized into 4 groups: 1) aerobic training; 2) aerobic training + strength training; 3) high intensity interval training; 4) control group. In phase 2, the training modality that has shown the most benefit on glycaemic control in phase 1 will be performed. During phase 2 there will be 3 groups: 1) 2 days/week training; 2) 3 days/week training; 3) 5 days/week training. All training sessions are adapted and specially designed for the prediabetes population.

Who provided:

In relation to the exercise intervention sessions, a sports professional will design and implement the sessions. The medical examination to assess whether each participant can perform physical exercise safely, will be performed by a physician.

How (mode of delivery; individual or group):

The intervention will be face-to-face, in small groups of 4-8 participants.

Where:

The exercise interventions will take place in the University of the Balearic Islands facilities, such as the cross-country circuit, the gymnasium, the swimming pool, and the multi-purpose rooms.

When and how much:

Face-to-face consultation will be at baseline and at 15 weeks, to perform the post intervention data collection consultation. During the 15 weeks of intervention, weekly face-to-face sessions will be held in small groups of 4-8 people.

Tailoring:

The intervention will be personalized in terms of the characteristics and preferences of the participants. In the case of the two interventions in which aerobic activity will be performed, given the characteristics of this activity, 4 alternatives will be offered to participants to choose 1 or 2. In the case of making adaptations to their characteristics, it means that if a person cannot perform a particular exercise due to lack of strength or because it produces some discomfort, the activity will be adapted.

Form saved

How well (planned):

GLYCEX study (GLYcaemic Control with EXercise)

Adherence to the study will be measuted by the atendance at the training sessions that make up the intervention.

Form saved
